# The effect of attendance in the Dutch breast cancer screening program on breast tumor characteristics among migrant women

**DOI:** 10.1016/j.breast.2023.03.008

**Published:** 2023-03-15

**Authors:** R.R.E. Dassen, S. Pelders, L. de Munck, A. Jager, M.J. Hooning, J.H. van Dam, B.A.M. Heemskerk-Gerritsen

**Affiliations:** a Erasmus MC Cancer Institute, Medical Oncology, Rotterdam, Netherlands; b Netherlands Comprehensive Cancer Organization, Research and Development, Utrecht, Netherlands; c Erasmus MC Cancer Institute, Oncological Surgery, Rotterdam, Netherlands

**Keywords:** Breast cancer, Screening, Migrants, Tumor characteristics, Incidence

## Abstract

**Background:**

In general, migrant women have a lower breast cancer (BC) incidence rate and higher BC mortality than autochthonous women. Further, migrant women show lower participation in the national BC screening program. To further investigate those aspects, we aimed to determine differences in incidence and tumor characteristics between autochthonous and migrant BC patients in Rotterdam, the Netherlands.

**Methods:**

We selected women diagnosed with BC in Rotterdam during 2012–2015 from the Netherlands Cancer Registry. Incidence rates were calculated by migrant status (i.e., women with or without migration background). Multivariable analyses revealed adjusted odds ratios (OR) and 95% confidence intervals (CI) on the association between migration status and patient and tumor characteristics, additionally stratified by screening attendance (yes/no).

**Results:**

In total 1372 autochthonous and 450 migrant BC patients were included for analysis. BC incidence was lower among migrants than among autochthonous women. Overall, migrant women were younger at BC diagnosis (53 vs. 64 years, p < 0.001), and had higher risks of positive lymph nodes (OR 1.76, 95% CI 1.33–2.33) and high grade tumors (OR 1.35, 95% CI 1.04–1.75). Especially non-screened migrant women had higher risk of positive nodes (OR 2.73, 95% CI 1.43–5.21). Among the subgroup of screened women, we observed no significant differences between migrant and autochthonous patients.

**Conclusion:**

Migrant women have lower BC incidence than autochthonous women, but diagnosis was more often at younger age and with unfavorable tumor characteristics. Attending the screening program strongly reduces the latter. Therefore, promotion of participation in the screening program is recommended.

## Highlights

• Breast cancer incidence is lower for migrant women than for autochthonous women.
• Among migrant women breast cancer diagnosis is more often at younger age.
• Higher risks of unfavorable tumor characteristics for migrant women.
• No differences in tumor characteristics when attending the screening program.

## 1 Introduction

Breast cancer (BC) is the most common type of cancer in women in developed countries. Approximately 15,000 women are diagnosed with invasive BC every year in the Netherlands. One out of seven women living in the Netherlands (14–15%) will develop BC during their life, and approximately 20% of women diagnosed with BC in the Netherlands will die from the disease [1].

Population-based BC screening results in earlier diagnosis of BC with better prognosis and consequently reduced BC-specific mortality [[2], [3], [4]]. However, not all women attend the screening program. In the Netherlands, where screening consists of biennial mammography for women aged 50–75 years and is free of costs, average attendance rate in the population-based BC screening program is around 80% [5]. Moreover, participation of women with a foreign background in first or second degree (hereafter referred to as ‘migrant’) is considerably lower than among autochthonous women (63% versus 83%, respectively) [6,7].

Prior research observed lower BC incidence rates for largest migrant groups in the Netherlands (i.e., Turkish, Moroccan, Surinamese, Indonesian) than for autochthonous women. However, mortality rates were higher for Moroccan and Surinamese migrants [[8], [9], [10]]. The observed mortality differences between autochthonous and migrant BC patients may be due to differences in patient and tumor characteristics at time of diagnosis (i.e., age, ethnicity, lifestyle, reproductive factors, tumor stage, histological grade, hormone and Her2neu receptor status), choice of therapy or a combination of factors [11,12]. While in general the incidence of advanced BC is higher in non-screened than in screened women, it is unclear so far whether this also applies for migrant women in the Netherlands [2].

Given the fact that in general every second citizen in Rotterdam has a foreign background in first or second degree and the low screening attendance rate among migrant women, the objective of this retrospective study is to investigate potential differences in incidence and tumor characteristics among autochthonous and migrant BC patients in Rotterdam, and the role of participation in the BC screening program in this regard.

## 2 Participants and methods

### 2.1 Study population

For this study we selected all women in Rotterdam diagnosed with BC (invasive in situ, excl. lobular carcinoma in situ) between January 1, 2012 and December 31, 2015. We used data from the Netherlands Cancer Registry (NCR), managed by the Netherlands Comprehensive Cancer Organization (IKNL). This data was linked to data from the Netherlands Breast Cancer Screening Registry. Linkage data identifies women screened between January 1, 2010 and December 31, 2015 to cover a period of at least 24 months before BC diagnosis, which was relevant for defining the screening status of BC.

We defined BC screening status as follows. Non-screened BC included cases of women who never attended the screening program. Among screened patients we distinguished between screen-detected BC being diagnosed within 12 months after the last screening round, and interval BC being diagnosed 12–24 months after a negative screening result. Additionally, we defined screening status as other BC for cases diagnosed at a screening interval longer than the scheduled 24 months, because those patients have been screened in the past, implying that they were familiar with the screening program.

Two groups of BC patients were defined: autochthonous and migrant patients (i.e., patients with a migration background in first or second degree). To identify migrant status a name-based approach was used based on surname in the NCR. If information about country of birth was accessible in NCR, migrant status was checked, as described before [13].

We excluded women whose migration background was unknown and women diagnosed with BC before the current diagnosis, since the latter may influence whether or not to participate the screening program. For women with synchronous BC, most advanced cancer was used for analysis.

### 2.2 Data collection

Since 1989 IKNL registers information about all new cases of cancer (including non-invasive tumors) in the Netherlands. Collected items are based on international guidelines (i.e., World Health Organization and International Association of Cancer Registries). Tumor stage is recorded according to TNM classification, seventh edition [14].

Numbers of new cases among the autochthonous and migrant population were acquired from the NCR dataset and numbers of population at risk from Statistics Netherlands [15]. For the current study we used data on patient and tumor characteristics, i.e., date of birth, sex, socio-economic status (SES) and migrant status, date of diagnosis, screening status, clinical TNM-stage, histological grade and receptor status; estrogen receptor (ER), progesterone receptor (PR), Human Epidermal growth factor Receptor 2 (HER2Neu). The SES variable was based on average income, educational status and number of unemployed in the geographical area a patient lives (4-digit postcode).

### 2.3 Population-based screening program in the Netherlands

The national BC screening program was implemented in the Netherlands in 1990, and is free of costs. Women aged 50–75 years receive an invitation every 2 years. Mammograms are independently assessed by two radiologists according to the Breast Imaging Reporting and Data System (BI-RADS) [16]. If examination is insufficient (BI-RADS 0) or suspected of malignancy (BI-RADS 4/5) a woman is referred for further diagnostic assessment [5].

### 2.4 Statistical analyses

Baseline patient and tumor characteristics were compared between autochthonous and migrant BC patients using chi-square tests for categorical variables and unpaired t-tests for continuous variables. Main outcomes of interest were standardized incidence rate, age at BC diagnosis, screening status and tumor characteristics (i.e., clinical TNM-stage, T-stage and N-stage, histological grade, ER status, PR status and Her2Neu status). For the purpose of this study, outcomes were divided into two groups representing more and less favorable characteristics. More specific, age at diagnosis >50 vs. <50 years, screen-detected vs. non-screened tumors, cTNM stage 0/I vs. II+, cT stage Tis/T1 vs. T2+, no lymph node involvement (cN negative) vs. lymph node involvement (cN positive), no metastasis (cM0) vs. metastasized disease (cM1), low grade (grade 1–2) vs. high grade (grade 3), ER positive vs. ER negative, PR positive vs. PR negative and HER2 negative vs. HER2 positive [11,12]. Age-specific incidence rates were calculated for each 10-year age group for autochthonous and migrant BC patients in Rotterdam using the female autochthonous and migrant population of Rotterdam as denominator. Age-standardized incidence rates (ASR) were calculated for each 10-year age group, using age distribution of the autochthonous population of Rotterdam as standard. Further, we calculated age-standardized incidence rate ratio's (ASRR) with 95% confidence intervals (CI) using the exact method and the autochthonous population of Rotterdam as reference.

To estimate the association between migration status and patient and tumor characteristics, univariable and multivariable (adjusted for age at diagnosis (as a continuous variable), year of diagnosis, SES and screening status) logistic regression analyses were used. Additionally, for patients in the age category that is eligible for attending the Dutch BC screening program (50–75 years) analyses were stratified for screening status. Additional analyses were executed for non-screened patients aged <50 years at time of diagnosis.

P-values were two-sided and a significance level of α = 0.05 was used. Statistical analyses were performed using IBM SPSS Statistics for Windows, Version 25.0. (IBM Corporation, Armonk, New York).

## 3 Results

Finally, we included 1822 women with BC diagnosed in the region of Rotterdam between 2012 and 2015, after excluding women with unknown migrant status (n = 98) and women diagnosed with BC before current diagnosis (n = 43). As shown in Table 1, 1372 patients (75.3%) were included in the autochthonous group and 450 patients in the migrant group (24.7%). Migrant women were younger at diagnosis than autochthonous women (median 53 vs. 64 years, p < 0.001), and more often under the age of 50 at time of diagnosis (33.6% versus 12.6%, p < 0.001). Percentage of interval tumors was higher in the autochthonous group than in the migrant group (12.0% vs. 6.7%, p = 0.013). Migrant patients more often had low SES (85.8% vs. 72.0% for autochthonous patients; p < 0.001).

**Table 1:** Patient and tumor characteristics of women diagnosed with breast cancer in Rotterdam between 2012 and 2015.

	Autochthonous		Migrant	
	N	%	N	%
**Total**	1372	75.3	450	24.7
**Median age at diagnosis**	64 (22–97)		53 (23–97)	
**Age**				
20-29	7	0.5	11	2.4
30-39	37	2.7	54	12.0
40-49	142	10.3	103	22.9
50-59	325	23.7	150	33.3
60-69	378	27.6	73	16.2
70-79	254	18.5	45	10.0
≥80	229	16.7	14	3.1
**50-year cut-off**				
<50 years	173	12.6	151	33.6
>50 years	1199	87.4	299	66.4
**Screening status**				
Screen-detected	439	32.0	161	35.8
Interval (≤24 months)	165	12.0	30	6.7
Other (>24 months)	86	6.3	30	6.7
Non-screened	682	49.7	229	50.9
**SES**				
Low (1–3)	986	72.0	386	85.8
Medium/High (4–10)	386	28.0	64	14.2
**cTNM stage**				
Stage 0/I	747	57.1	231	54.5
Stage II+	562	42.9	193	45.5
*Unknown*	*63*		*26*	
**cT stage**				
TIS/T1	802	60.8	263	61.6
T2+	516	39.2	164	38.4
*Unknown*	*54*		*23*	
**cN stage**				
N negative	1106	82.2	322	72.4
N positive	240	17.8	123	27.6
*Unknown*	*26*		*5*	
**cM stage**				
M0	1296	92.7	426	95.1
M1	72	5.3	22	4.9
*Unknown*	*4*		*2*	
**Histological grade**				
Low grade (1–2)	790	68.5	220	57.7
High grade (3)	360	31.5	161	42.3
*Unclear/Not applicable*	*219*		*69*	
ER status*				
ER positive	991	83.7	289	77.9
ER negative	193	16.3	82	22.1
*Unknown*	*188*		*79*	
PR status*				
PR positive	815	69.0	249	67.1
PR negative	369	31.0	122	32.9
*Unknown*	*188*		*79*	
HER2 status**				
HER2 positive	136	11.7	69	18.9
HER2 negative	1025	88.3	297	81.1
*Unknown*	*188*		*79*	
**Molecular subtype**				
HR+/HER2-	881	75.9	238	65.0
HR+/HER2+	100	8.6	50	13.7
HR-/Her2+	36	3.1	19	5.2
Triple Negative	144	12.4	59	16.1
*Unknown*	*188*		*79*	

*The cut-off point for ER/PR positivity is at least 10% of immunoreactive staining of the tumor cells.

**The tumor is considered HER2 positive if the immunohistochemistry score is 3+ or 2+ with amplification of the HER2 gene as assessed with an in-situ hybridization test.

Abbreviations: SES, socio-economic status; c, clinical; ER, estrogen receptor; PR, progesterone receptor; HER2, Human Epidermal growth factor Receptor 2; HR, hormone receptor; HR+/HER2-, ER and/or PR positive and HER2 negative; HR+/HER2+, ER and/or PR positive and HER2 positive; HR-/HER2+, ER and PR negative and HER2 positive; Triple Negative, ER, PR and HER2 negative.

Migrant women were more often diagnosed with a tumor with less favorable characteristics than autochthonous women; i.e., lymph node involvement (27.6% vs. 17.8%, p < 0.001), grade 3 (42.3% vs. 31.5%, p < 0.001), ER-negative (22.1% vs. 16.3%, p = 0.011), and HER2-positive (18.9% vs. 11.7%, p = 0.002). Migrant women were less often diagnosed with HR+/HER2-disease (65.0% vs. 75.9% for autochthonous patients).

### 3.1 Incidence

**Fig. 1:**
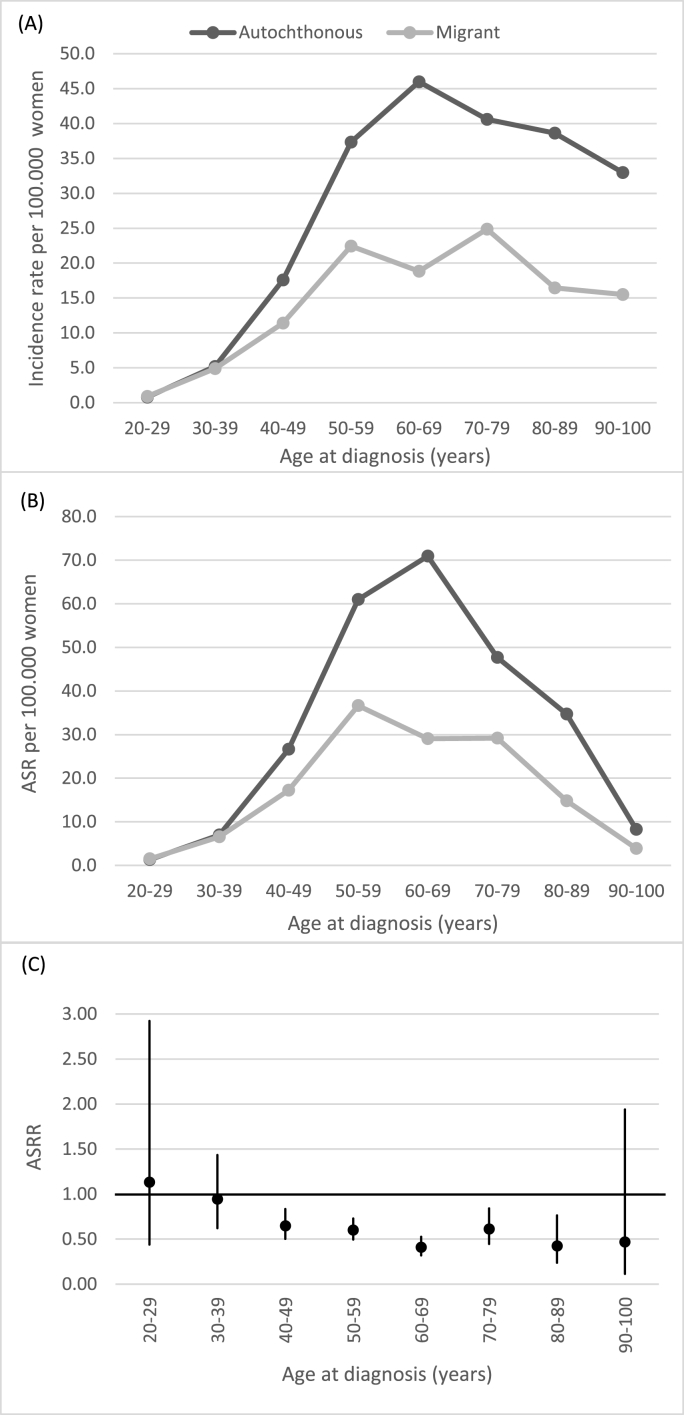
(A) Age-specific incidence rate per 100.000 women. (B) Age-standardized incidence rate per 100.000 women (ASR). (C) Age-standardized incidence rate ratio's (ASRR) and 95% confidence intervals, autochthonous women as baseline.

Fig. 1a shows age-specific incidence of BC patients in autochthonous and migrant groups. Highest incidence rates for autochthonous women were observed in age-group 60–69 years. For migrants peak incidences were at 50–59 and 70–79 years. As demonstrated in Fig. 1b, peak ASR's were observed at 60–69 years for autochthonous patients and at 50–59 years for migrant women. Overall, migrants had a lower ASR than autochthonous women (13.89 vs. 25.75, ASRR 0.54, 95% CI 0.48–0.60, p < 0.001). However, among younger women (<40 years at diagnosis) age-standardized incidence rates were similar for migrant and autochthonous women (Fig. 1b). In age-groups above 40 years, the ASRR's were in favor of migrants (Fig. 1c).

### 3.2 Association between migrant status and tumor characteristics

**Fig. 2:**
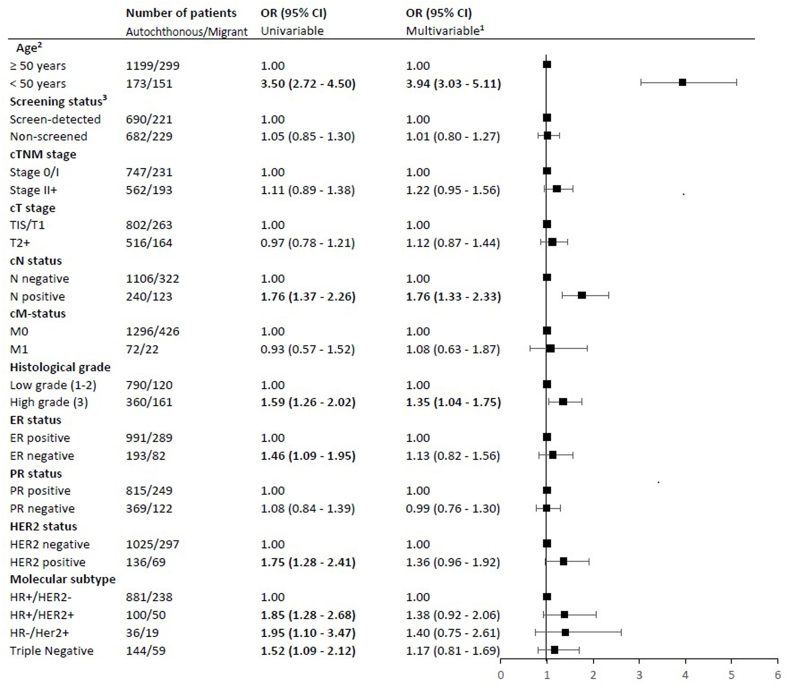
Association between migrant status and patient and tumor characteristics. OR (95% CI) = odds ratio (95% confidence interval). ^1^Adjusted for age, year of diagnosis, SES and screening status. ^2^Adjusted for year of diagnosis, SES and screening status. ^3^Adjusted for age, year of diagnosis and SES. Abbreviations: c, clinical; ER, estrogen receptor; PR, progesterone receptor; HER2, Human Epidermal growth factor Receptor 2; HR, hormone receptor; SES, socio-economic status.

As shown in Fig. 2, migrant patients had higher risks of being diagnosed with BC with less favorable characteristics than autochthonous women. After adjustment migrant women had higher risk of being younger than 50 years of age at diagnosis (adjusted OR 3.94, 95% CI 3.03–5.11). Further, migrants were almost two times more likely to be diagnosed with positive lymph nodes (adjusted OR 1.76, 95% CI 1.33–2.33) and had an increased risk for high grade disease (adjusted OR 1.35, 95% CI 1.04–1.75).

### 3.3 Screened BC patients aged 50–75 years

**Fig. 3:**
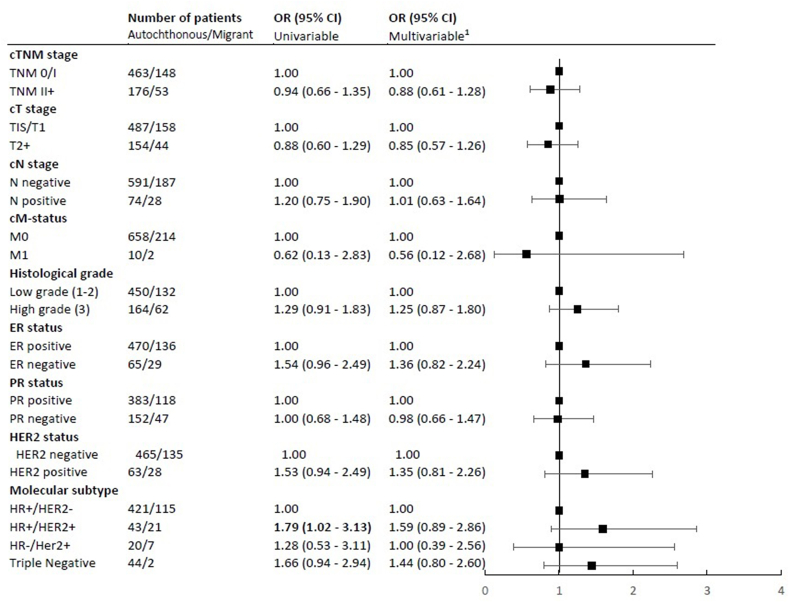
Association between migrant status and tumor characteristics for women, aged 50–75 years, diagnosed with breast cancer in Rotterdam between 2012 and 2015 who did attend the screening program. OR (95% CI) = odds ratio (95% confidence interval). ^1^Adjusted for age, year of diagnosis and SES. Abbreviations: c, clinical; ER, estrogen receptor; PR, progesterone receptor; HER2, Human Epidermal growth factor Receptor 2; HR, hormone receptor; SES, socio-economic status.

Among screened women, 672 patients (72.4%) were autochthonous and 218 patients (23.5%) were migrant (Supplementary Table S1). Migrant patients were younger at time of diagnosis than autochthonous patients (59 years vs. 62 years, p < 0.001). Migrant patients more often had a low SES and were less often diagnosed with an interval tumor. We observed no significant associations between migrant status and tumor characteristics (Fig. 3).

### 3.4 Non-screened BC patients aged 50–75 years

The subgroup of women who were invited for screening but did not attend, consisted of 234 autochthonous women (79.9%) and 59 migrant women (20.1%; Supplementary Table S2). Migrant women were younger at BC diagnosis than autochthonous women (55 years vs. 61.5 years, p = 0.002). Migrant non-screened women had higher proportions of unfavorable tumor characteristics than non-screened autochthonous women of the same age-group, i.e., positive lymph nodes (39.7% vs. 20.5%, p = 0.003), grade 3 (60.0% vs. 35.9%, p = 0.003), ER-negative (38.0% vs 18.8%, p = 0.003) and PR-negative (48.0% vs 31.5%, p = 0.027). Additionally, migrants in this subgroup were less often diagnosed with HR+/HER2-disease (54.2% vs. 74.2%) and more often with triple negative (TN)-disease (33.3% vs. 14.8%, p = 0.009).

**Fig. 4:**
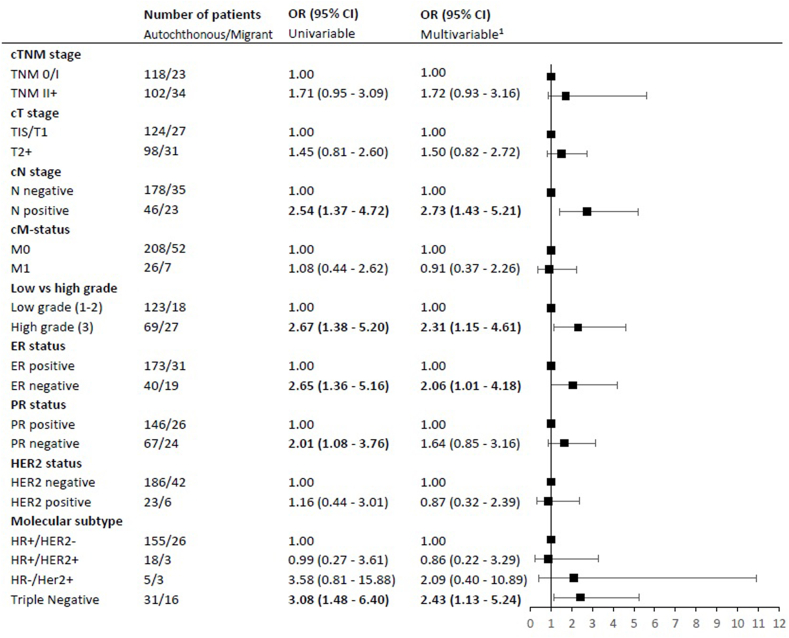
Association between migrant status and tumor characteristics for non-screened women, aged 50–75 years, diagnosed with breast cancer in Rotterdam between 2012 and 2015. OR (95% CI) = odds ratio (95% confidence interval). ^1^Adjusted for age, year of diagnosis and SES. Abbreviations: c, clinical; ER, estrogen receptor; PR, progesterone receptor; HER2, Human Epidermal growth factor Receptor 2; HR, hormone receptor; SES, socio-economic status.

As shown in Fig. 4, among non-screened 50–75 aged patients, being migrant was associated with higher risks of being diagnosed with N-positive disease (adjusted OR 2.73, 95% CI 1.43–5.21), high grade disease (adjusted OR 2.31, 95% CI 1.15–4.61), ER-negative disease (adjusted OR 2.06, 95% CI 1.01–4.18), and a TN tumor (OR 2.43, 95% CI 1.13–5.24).

### 3.5 BC patients aged <50 years

Of women aged <50 years who were not screened given their age, 173 were autochthonous (53.4%) and 151 had a migrant background (46.6%; Supplementary Table S3). Compared to autochthonous patients, migrant women were younger at time of diagnosis (median 41 vs. 43 years, p = 0.015), more often had a low SES score (81.5% vs. 60.1%, p < 0.001), and more often were diagnosed with positive lymph nodes (40.0% vs. 24.6%, p = 0.003).

**Fig. 5:**
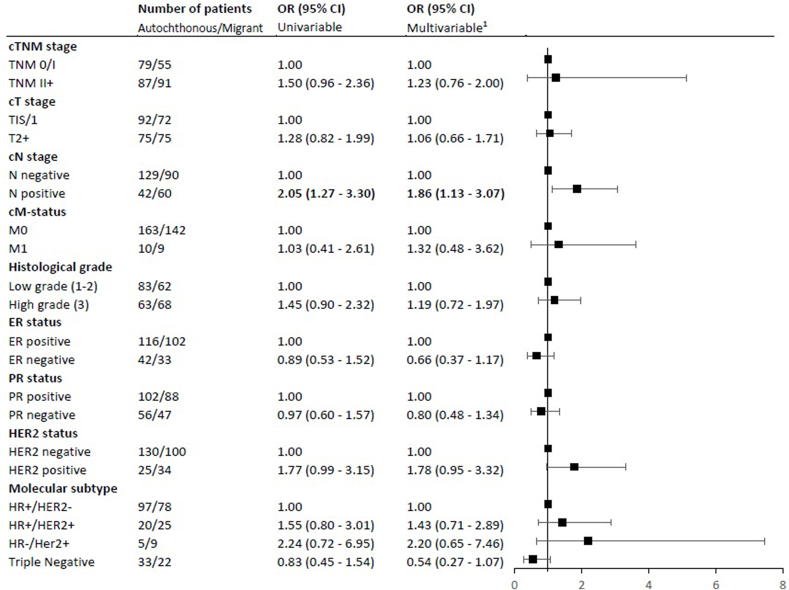
Association between migrant status and patient and tumor characteristics for women aged <50 years, not eligible for screening, diagnosed with breast cancer in Rotterdam between 2012-2015. OR (95% CI) = odds ratio (95% confidence interval). ^1^Adjusted for age, year of diagnosis and SES. Abbreviations: c, clinical; ER, estrogen receptor; PR, progesterone receptor; HER2, Human Epidermal growth factor Receptor 2; HR, hormone receptor; SES, socio-economic status.

As demonstrated in Fig. 5, for unscreened patients <50, being migrant was associated with a higher risk of being diagnosed with positive lymph nodes (adjusted OR 1.86, 95% CI 1.13–3.07).

## 4 Discussion

This retrospective study shows lower BC incidence among migrant women than among autochthonous women living in Rotterdam. However, migrants had higher risks of being diagnosed with BC with more unfavorable characteristics, especially younger age at BC diagnosis, and more often lymph node involvement and high tumor grade. Remarkably, among patients who did attend the BC screening program, migrants still had lower incidence and were younger at BC diagnosis, but migrant status was no longer associated with higher risks of other unfavorable tumor characteristics.

Lower BC incidence rates for migrants than for autochthonous women has been reported before [8,9,17]. Genetic factors may play a role in these different rates of BC incidence in different populations. Nonetheless, the majority of risk variation is still believed to be due to different lifestyle and environmental factors, such as age at first birth, parity, age at menarche, duration of breastfeeding, BMI and hormone therapy use [18].

In the current study, for women aged <50 years the incidence rates were similar for migrant and autochthonous women. Women in this age group are predominantly second-generation migrants, because of labor migration of their parents to the Netherlands from 1964 till 1973 and the post-independence migration from Suriname since 1975. The finding of similar incidence rates for younger migrant and autochthonous women may support earlier hypotheses that incidence rates gradually converge to levels of the new host population and migrants undergo changes in BC risk after change of residence [17,19].

Our finding of migrant BC patients being younger at time of diagnosis than autochthonous women are in line with results from previous studies [[20], [21], [22], [23], [24], [25], [26]]. Similar to Jack et al. [23], we found a significant lower median age at time of diagnosis for migrant women, with a difference of ±11 years. Noteworthy, the majority of migrant women in Rotterdam are from Surinamese, Turkish or Moroccan descent [27]. The mean age at BC diagnosis in these countries is known to be lower than in the Netherlands [[28], [29], [30]]. Interestingly, Turkey and Morocco have lowered the starting age of the screening program to the age of 40, because of the high proportions of BCs diagnosed under the age of 50 [28,29]. Since the percentage of migrants in the Netherlands is expected to rise, our findings could contribute to the discussion of lowering the starting age of the BC screening program or to the discussion on personalized screening [31,32]. Studies have shown that lowering the screening reduces BC mortality and is cost-effective [32,33].

Current findings confirm previous observations on migrant patients more often being diagnosed with poorly differentiated and/or ER negative BC than autochthonous patients [21,22,25]. The etiology of higher risks of these unfavorable tumor characteristics remains unclear. Inherited genomic profiles, which vary in different ethnic groups, may predict the development of a specific tumor biology [34,35]. Literature also describes positive associations between high histologic grades and unfavorable characteristics, such as positive nodes and negative ER status [36]. Indeed, migrant women in our study more often had high grade tumors as well as more often positive nodes. However, lifestyle and environmental conditions, such as reproductive factors and physique could also play a role. Parous women, young age at first birth and breastfeeding was associated with a reduced risk of developing BC, especially of hormone receptor (HR) positive type [37]. This could explain lower proportion of HR-positive tumors in migrants, since culturally they are younger at birth of the first child and breastfed more often, for a longer period of time [38,39]. Furthermore, being taller has been be associated with more HR-positive BC [40]. Since autochthonous Dutch people belong to the tallest populations of the world [41], this also could partly explain the higher proportion of HR-positive disease among autochthonous patients in the current study.

Our results indicate that BC screening may diminish the observed differences in tumor characteristics between autochthonous and migrant BC patients. While we observed in the non-screened group higher risks of lymph node positive, high grade and ER-negative tumors for migrants than for autochthonous patients, this difference disappeared in the screen-related group. In addition to less lymph node involvement, screen-detected cancers may more often have favorable tumor grade and expression of hormone receptors, which might be explained by an association between clinicopathologic features and molecular profiles [42].

Screening has been proven to be beneficial to reduce treatment-related burdens and mortality associated with BC [2]. Although migrant women have lower BC incidence, our study contributes to the evidence that they have higher risk of being diagnosed at younger age and with unfavorable tumor characteristics. These characteristics are considered important prognostic factors [11,12,36]. Therefore, earlier diagnosis of aggressive tumors may provide significant health gain for these patients. To increase the attendance rate of migrant women into the BC screening program more research on information, awareness, motivation, ability and barriers is needed. Understanding of determinants of participation for subpopulations and efforts to influence them, will also provide benefits for attendance into other population-based cancer screening programs (e.g., cervical and colorectal). In addition, this might also affect participation rate in other preventive health programs, such as the national vaccination program and COVID-19 vaccination [43].

To our knowledge this is the first study in the Netherlands to analyze differences in tumor stage and biology between autochthonous and migrant BC patients and to determine the influence of screening on these differences. However, some limitations must be considered when interpreting the results of this study. First, analyzing ethnic subgroups may be preferable, because of known diversity in environmental and genetic BC risk factors between western and non-western migrants [18,19,44]. Unfortunately, specific information on ethnicity was unavailable due to the European General Data Protection Regulation [45]. Second, the definition of migrant which was based on surname by the NCR. However, some migrants have one migrant parent and one Dutch parent, and may therefore carry a Dutch surname. Fortunately, this potential misclassification may have led to an underestimation rather than overestimation of actual BC incidence and risk for more unfavorable tumor characteristics among migrants, thus not influencing our conclusions. Moreover, we expect misclassification based on surname to be confined to second-generation migrants, being the younger group of patients. Since this group is in minority in our study, we expect potential underestimation will be limited. Lastly, in analysis stratified by screening status, one must consider the smaller sample size of migrant women with aggressive tumor features, which could result in imprecision of results.

When using routinely collected data, little information on personal characteristics which can affect tumor characteristics (i.e., ethnicity, duration since migration, age at migration, physique, reproductive and hormonal factors) is available. Therefore, for future research, including this information will allow a better understanding of differences between autochthonous and migrant BC patients.

In conclusion, BC incidence is almost two times lower among migrant women than among autochthonous women in Rotterdam. However, migrant women are younger at BC diagnosis and have more unfavorable tumor characteristics than autochthonous women, such as lymph node involvement and high tumor grade. This is especially true for non-screened migrant women. Attending the BC screening program reduces differences in risk of unfavorable tumor characteristics between migrant and autochthonous BC patients. Therefore, promoting participation in the national BC screening program is important to increase attendance rate for migrant women, which will lead to earlier BC detection and subsequent improved BC-specific survival.

## Funding information

This research did not receive any specific grant from funding agencies in the public, commercial, or not-for-profit sectors.

## Ethics approval and consent to participate

The registry data used in this study was made available to us in an anonymized form by the Netherlands Cancer Registry. In the Netherlands, studies using anonymized registry data do not require specific consent nor approval by an ethics committee. The study was performed in accordance with the Declaration of Helsinki.

## Additional information

### Consent for publication

This paper does not contain any individual-level data.

## Declaration of competing interest

The authors have no conflicts of interest to disclose.

## Data Availability

The data that support the findings of our study are available from the corresponding author upon reasonable request.
