# Supplementary material for: The effect of attendance in the Dutch breast cancer screening program on breast tumor characteristics among migrant women
Source: Breast. 2023 Mar 15;69:290–8. doi: 10.1016/j.breast.2023.03.008 (PMC10034141; doi:10.1016/j.breast.2023.03.008)
Supplement: Multimedia component 1 [file mmc1.docx]

**Supplementary Table S1. Patient and tumor characteristics of women aged 50-75 years at screening invitation, diagnosed with breast cancer in Rotterdam between 2012-2015 who did attend the screening program**

|  | **Autochthonous** |  | **Migrant** |  |
| --- | --- | --- | --- | --- |
|  | N | % | N | % |
| **Total** | 672 | 72.4 | 218 | 23.5 |
| **Median age at diagnosis** | 62 (49-76) |  | 59 (49-76) |  |
| **Age** |  |  |  |  |
| 49-59 | 238 | 35.4 | 126 | 57.8 |
| 60-69 | 294 | 43.8 | 63 | 28.9 |
| 70-76 | 140 | 20.8 | 29 | 13.3 |
| **Screening status** |  |  |  |  |
| Screen-detected | 439 | 65.3 | 161 | 73.9 |
| Interval (< 24 months) | 163 | 24.3 | 30 | 13.8 |
| Other (> 24 months) | 70 | 10.4 | 27 | 12.4 |
| Non-screened | 0 | 0.0 | 0 | 0.0 |
| **SES** |  |  |  |  |
| Low (1-3) | 460 | 68.5 | 193 | 88.5 |
| Medium/High (4-10) | 212 | 31.5 | 25 | 11.5 |
| **cTNM stage** |  |  |  |  |
| Stage 0/I | 463 | 72.5 | 148 | 73.6 |
| Stage II+ | 176 | 27.5 | 53 | 26.4 |
| *Unknown* | *33* |  | *17* |  |
| **cT stage** |  |  |  |  |
| TIS/T1 | 487 | 76.0 | 158 | 78.2 |
| T2+ | 154 | 24.0 | 44 | 21.8 |
| *Unknown* | *31* |  | *16* |  |
| **cN stage** |  |  |  |  |
| N negative | 591 | 88.9 | 187 | 87.0 |
| N positive | 74 | 11.1 | 28 | 13.0 |
| *Unknown* | *7* |  | *3* |  |
| **cM stage** |  |  |  |  |
| M0 | 658 | 98.5 | 214 | 99.1 |
| M1 | 10 | 1.5 | 2 | 0.9 |
| *Unknown* | *4* |  | *2* |  |
| **Histological grade** |  |  |  |  |
| Low grade (1-2) | 450 | 73.3 | 132 | 68.0 |
| High grade (3) | 164 | 26.7 | 62 | 32.0 |
| *Unclear/Not applicable* | *58* |  | *24* |  |
| **ER status** |  |  |  |  |
| ER positive | 470 | 87.9 | 136 | 86.6 |
| ER negative | 65 | 12.1 | 29 | 13.4 |
| *Unknown* | *137* |  | *53* |  |
| **PR status** |  |  |  |  |
| PR positive | 383 | 71.6 | 118 | 71.5 |
| PR negative | 152 | 28.4 | 47 | 28.5 |
| *Unknown* | *137* |  | *53* |  |
| **HER2 status** |  |  |  |  |
| HER2 positive | 63 | 10.9 | 28 | 17.2 |
| HER2 negative | 465 | 89.1 | 135 | 82.8 |
| *Unknown* | *144* |  | *55* |  |
| **Molecular subtype** |  |  |  |  |
| HR+/HER2- | 421 | 79.7 | 115 | 79.3 |
| HR+/HER2+ | 43 | 8.2 | 21 | 14.5 |
| HR-/Her2+ | 20 | 3.8 | 7 | 4.8 |
| Triple Negative | 44 | 8.3 | 2 | 1.4 |
| *Unknown*  Abbreviations: SES, socio-economic status; c, clinical; ER, estrogen receptor; PR, progesterone receptor; HER2, Human Epidermal growth factor Receptor 2; HR, hormone receptor. | *144* |  | *55* |  |

**Supplementary Table S2. Patient and tumor characteristics of non-screened women, aged 50-75 years at screening invitation, diagnosed with breast cancer in Rotterdam between 2012 and 2015**

|  | **Autochthonous** |  | **Migrant** |  |
| --- | --- | --- | --- | --- |
|  | N | % | N | % |
| **Total** | 234 | 79.9 | 59 | 20.1 |
| **Median age at diagnosis** | 61.50 (49-76) | | 55 (49-76) |  |
| **Age** |  |  |  |  |
| 49-59 | 100 | 42.7 | 41 | 69.5 |
| 60-69 | 84 | 35.9 | 10 | 16.9 |
| 70-76 | 50 | 21.4 | 8 | 13.6 |
| **SES** |  |  |  |  |
| Low (1-3) | 182 | 77.8 | 54 | 91.5 |
| Medium/High (4-10) | 52 | 22.2 | 5 | 8.5 |
| **cTNM stage** |  |  |  |  |
| Stage 0/I | 118 | 53.6 | 23 | 40.4 |
| Stage II+ | 102 | 46.4 | 34 | 59.6 |
| *Unknown* | *14* |  | *2* |  |
| **cT stage** |  |  |  |  |
| TIS/T1 | 124 | 55.9 | 27 | 46.6 |
| T2+ | 98 | 44.1 | 31 | 53.4 |
| *Unknown* | *12* |  | *1* |  |
| **cN stage** |  |  |  |  |
| N negative | 178 | 79.5 | 35 | 60.3 |
| N positive | 46 | 20.5 | 23 | 39.7 |
| *Unknown* | *10* |  | *1* |  |
| **cM stage** |  |  |  |  |
| M0 | 208 | 88.9 | 52 | 88.1 |
| M1 | 26 | 11.1 | 7 | 11.9 |
| *Unknown* | *0* |  | *0* |  |
| **Histological grade** |  |  |  |  |
| Low grade (1-2) | 123 | 64.1 | 18 | 40.0 |
| High grade (3) | 69 | 35.9 | 27 | 60.0 |
| *Unclear/Not applicable* | *42* |  | *14* |  |
| **ER status** |  |  |  |  |
| ER positive | 173 | 81.2 | 31 | 62.0 |
| ER negative | 40 | 18.8 | 19 | 38.0 |
| *Unknown* | *21* |  | *9* |  |
| **PR status** |  |  |  |  |
| PR positive | 146 | 68.5 | 26 | 52.0 |
| PR negative | 67 | 31.5 | 24 | 48.0 |
| *Unknown* | *21* |  | *9* |  |
| **HER2 status** |  |  |  |  |
| HER2 positive | 23 | 11.0 | 6 | 12.5 |
| HER2 negative | 186 | 89.0 | 42 | 87.5 |
| *Unknown* | *21* |  | *9* |  |
| **Molecular subtype** |  |  |  |  |
| HR+/HER2- | 155 | 74.2 | 26 | 54.2 |
| HR+/HER2+ | 18 | 8.6 | 3 | 6.3 |
| HR-/Her2+ | 5 | 2.4 | 3 | 6.3 |
| Triple Negative | 31 | 14.8 | 16 | 33.3 |
| *Unknown* | *21* |  | *9* |  |

Abbreviations: SES, socio-economic status; c, clinical; ER, estrogen receptor; PR, progesterone receptor; HER2, Human Epidermal growth factor Receptor 2; HR, hormone receptor.

**Supplementary Table S3. Patient and tumor characteristics of women aged <50 years, not eligible for screening, diagnosed with breast cancer in Rotterdam between 2012-2015**

Abbreviations: SES, socio-economic status; c, clinical; ER, estrogen receptor; PR, progesterone receptor; HER2, Human Epidermal growth factor Receptor 2; HR, hormone receptor.

|  | **Autochthonous** |  | **Migrant** |  |
| --- | --- | --- | --- | --- |
|  | N | % | N | % |
| **Total** | 173 | 53.4 | 151 | 46.6 |
| **Median age at diagnosis** | 43 (22 - 49) |  | 41 (23 - 49) |  |
| **Age** |  |  |  |  |
| 20-29 | 7 | 4.0 | 11 | 7.3 |
| 30-39 | 37 | 21.4 | 54 | 35.8 |
| 40-49 | 129 | 74.6 | 86 | 57.0 |
| **SES** |  |  |  |  |
| Low (1-3) | 104 | 60.1 | 123 | 81.5 |
| Medium/High (4-10) | 69 | 39.9 | 28 | 18.5 |
| **cTNM stage** |  |  |  |  |
| Stage 0/I | 79 | 47.6 | 55 | 37.7 |
| Stage II+ | 87 | 52.4 | 91 | 62.3 |
| *Unknown* | *7* |  | *5* |  |
| **cT stage** |  |  |  |  |
| TIS/T1 | 92 | 55.1 | 72 | 49.0 |
| T2+ | 75 | 44.9 | 75 | 51.0 |
| *Unknown* | *6* |  | *4* |  |
| **cN stage** |  |  |  |  |
| N negative | 129 | 75.4 | 90 | 60.0 |
| N positive | 42 | 24.6 | 60 | 40.0 |
| *Unknown* | *2* |  | *1* |  |
| **cM stage** |  |  |  |  |
| M0 | 163 | 94.2 | 142 | 94.0 |
| M1 | 10 | 5.8 | 9 | 6.0 |
| *Unknown* | *0* |  | *0* |  |
| **Histological grade** |  |  |  |  |
| Low grade (1-2) | 83 | 56.8 | 62 | 47.7 |
| High grade (3) | 63 | 43.2 | 68 | 52.3 |
| *Unclear/Not applicable* | *27* |  | *21* |  |
| **ER status** |  |  |  |  |
| ER positive | 116 | 73.4 | 102 | 75.6 |
| ER negative | 42 | 26.6 | 33 | 24.4 |
| *Unknown* | *15* |  | *16* |  |
| **PR status** |  |  |  |  |
| PR positive | 102 | 64.6 | 88 | 65.2 |
| PR negative | 56 | 35.4 | 47 | 34.8 |
| *Unknown* | *15* |  | *16* |  |
| **HER2 status** |  |  |  |  |
| HER2 positive | 25 | 16.1 | 34 | 25.4 |
| HER2 negative | 130 | 83.9 | 100 | 74.6 |
| *Unknown* | *15* |  | *16* |  |
| **Molecular subtype** |  |  |  |  |
| HR+/HER2- | 97 | 62.6 | 78 | 58.2 |
| HR+/HER2+ | 20 | 12.9 | 25 | 18.7 |
| HR-/Her2+ | 5 | 3.2 | 9 | 6.7 |
| Triple Negative | 33 | 21.3 | 22 | 16.4 |
| *Unknown* | *15* |  | *16* |  |
